# An assessment of the rescue action of resveratrol in *parkin* loss of function-induced oxidative stress in *Drosophila melanogaster*

**DOI:** 10.1038/s41598-022-07909-7

**Published:** 2022-03-10

**Authors:** Adeola O. Adedara, Ayoade D. Babalola, Flora Stephano, Ifeoluwa O. Awogbindin, James O. Olopade, João B. T. Rocha, Alexander J. Whitworth, Amos O. Abolaji

**Affiliations:** 1 Drosophila Laboratory, Molecular Drug Metabolism and Toxicology Unit, Department of Biochemistry, College of Medicine, University of Ibadan, Ibadan, Nigeria; 2 Cancer Research and Molecular Biology Laboratory, Department of Biochemistry, College of Medicine, University of Ibadan, Ibadan, Nigeria; 3 Department of Zoology and Wildlife Conservation, College of Natural and Applied Sciences, University of Dar es Salaam, Dar es Salaam, Tanzania; 4 Department of Veterinary Anatomy, Faculty of Veterinary Medicine, University of Ibadan, Ibadan, Nigeria; 5 Department of Biochemistry and Molecular Biology, Federal University of Santa Maria, R/S, Camobi, Santa Maria, Brazil; 6 MRC Mitochondria Biology Unit, University of Cambridge, Cambridge, UK

**Keywords:** Biochemistry, Neuroscience, Stress and resilience

## Abstract

Loss-of-function mutations in *parkin* is associated with onset of juvenile Parkinson’s disease (PD). Resveratrol is a polyphenolic stilbene with neuroprotective activity. Here, we evaluated the rescue action of resveratrol in *parkin* mutant *D. melanogaster*. The control flies (*w*^1118^) received diet-containing 2% ethanol (vehicle), while the PD flies received diets-containing resveratrol (15, 30 and 60 mg/kg diet) for 21 days to assess survival rate. Consequently, similar treatments were carried out for 10 days to evaluate locomotor activity, oxidative stress and antioxidant markers. We also determined mRNA levels of *Superoxide dismutase 1 *(*Sod1, an antioxidant gene*) and *ple,* which encodes tyrosine hydroxylase, the rate-limiting step in dopamine synthesis. Our data showed that resveratrol improved survival rate and climbing activity of PD flies compared to untreated PD flies. Additionally, resveratrol protected against decreased activities of acetylcholinesterase and catalase and levels of non-protein thiols and total thiols displayed by PD flies. Moreover, resveratrol mitigated against *parkin* mutant-induced accumulations of hydrogen peroxide, nitric oxide and malondialdehyde. Resveratrol attenuated downregulation of *ple* and *Sod1* and reduction in mitochondrial fluorescence intensity displayed by PD flies. Overall, resveratrol alleviated oxidative stress and locomotor deficit associated with *parkin* loss-of-function mutation and therefore might be useful for the management of PD.

## Introduction

Parkinson's disease (PD) is an idiopathic and neurodegenerative disease. The hallmark of PD is depletion in dopamine level, due to dopaminergic neuronal loss in the substantia nigra pars compacta and striatum, leading to movement disorder^1,2^. Apart from the environmentally-induced sporadic form, mutations in several genes such as SNCA, PRKN, ATP13A2, DJ-1, LRRK2 and *Pink1* have been linked with the familial form of PD^3,4^. Therefore, most PD incidences are sporadic, with only about 10–15% being familial cases^5^. In addition, 50% of all autosomal recessive familial or juvenile cases and nearly 15% of sporadic cases exhibit mutations in *Parkin* gene^6^. Indeed, mutations in *Parkin* gene are the second most frequent genetic causes of PD^7^. They occur in both early-onset familiar and sporadic forms of PD, with patients displaying clinical features of early and late parkinsonism^8^. The *Parkin* gene is composed of twelve exons that encode a 465 amino acid length protein (Parkin) with N-terminal ubiquitin-like motif, in-between ring-finger (IBR) domain, and C-terminal two-RING finger motif. The C-terminal motif possesses the ubiquitin ligase activity, whereas the loss of ubiquitin ligase activity in Parkin has been implicated in the pathogenicity of PD^7^.

*Parkin* is an E3 ubiquitin ligase and a downstream substrate of PTEN-induced kinase 1 (*Pink1)*. Together with *Pink1* and ubiquitin, *Parkin* maintains mitochondrial integrity during mitochondrial stress by ubiquitinating several outer mitochondrial membrane protein targets. Consequently, this leads to sequestration and mitophagy of depolarized or damaged mitochondria^4^. Apart from mitophagy, *Parkin* is also involved in mitochondrial biogenesis and dynamics leading to mitochondrial quality control system. Therefore, the homeostatic roles of mitochondria in energy-dependent tissue function and health, are through the quality control of proteins, partly involving *Parkin*^9^. The loss-of-function mutation of *Parkin* causes accumulation of depolarized mitochondria in energy-dependent tissues, such as muscles and brains^10^. This eventually induces mitochondrial dysfunctions, oxidative stress and inflammation^11^. Indeed, muscular and neurodegenerations, which are phenotypic features of PD, have been shown through *Parkin* knockout in different animal models^8^.

The loss-of-function mutations in Drosophila PARK2 (*parkin*) ortholog, causes PD-like phenotypes such as shortened life span, locomotor deficit^12,13^ and dopaminergic neuronal loss^14^ due to dysfunctional mitochondrial-mediated oxidative stress^15,16^. Drosophila has made it possible to carry out genetic manipulations to generate numerous PD models such as the expression of human genes with pathogenic mutations or targeted mutation of conserved orthologs^17^. Thus, *D. melanogaster* does not only provide a unique opportunity for understanding the mechanism of pathogenesis of PD, but also help in identifying targets for therapies.

Currently, pharmacological drugs employed in the management of PD, such as levodopa, dopamine agonist or monoamine oxidase B inhibitors, only treat the symptoms. Of note, the beneficial effects of levodopa, the most commonly used conventional drug, wears out over time and its clinical value declines as the disease advances. There are also major side-effects such as dyskinesia, swelling and impulse control disorders noticed in PD patients^18^. Owing to the lack of effective therapy in the management of PD, there may be a dramatic increase in the incidence of PD patients in the coming decades.

Oxidative stress has been implicated in the pathogenesis of PD. In healthy state, the body maintains a balance between oxidative stress and antioxidant status in order to ensure survival^19^. Thus, in order to reduce oxidative stress associated with PD, compounds possessing anti-oxidative property are currently being promoted as potential co-adjuvant molecules in the treatment of PD^20,21^. Truly, there is a growing line of evidence indicating that *parkin* mutant flies have been rescued with antioxidant-rich supplements and natural compounds^22–24^.

Natural polyphenols are promising medicinal and pharmaceutical compounds for the management of PD due to their high antioxidant content and low toxic properties. Resveratrol is a stilbene found in several plants such as grapes, berries and peanuts^25^. It has both anti-inflammatory and antioxidant^26^ properties. We have previously reported the protective role of resveratrol in 1-methyl-4-phenyl-1,2,3,6-tetrahydropyridine (MPTP)-induced model of PD^27^. Here, we hypothesized that resveratrol could serve as a therapeutic agent in Drosophila *parkin* mutant model of PD. In this regard, we sought to validate the potential therapeutic treatment of oxidative stress symptoms associated with *parkin* loss-of-function mutation in *D. melanogaster.*

## Results

### Resveratrol improves survival rate of *parkin* mutant *D. melanogaster*

**Figure 1:**
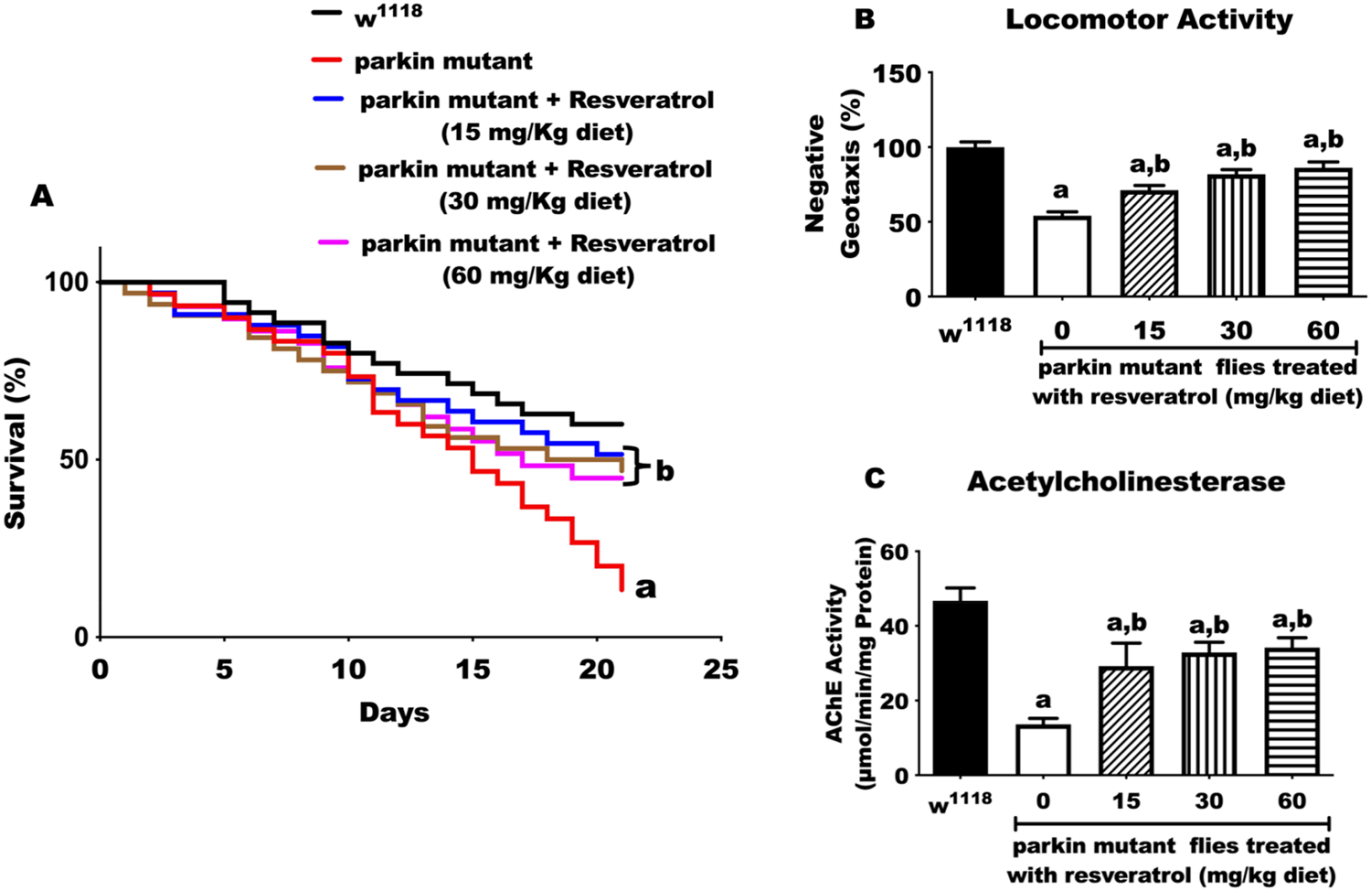
Effects of resveratrol on survival rate, locomotor (climbing) activity and acetylcholinesterase concentration of PD *Drosophila melanogaster*. **(A)** 21 days survival rate, **(B)** locomotor (climbing) activity and **(C)** acetylcholinesterase concentration of control and PD flies fed with diet-containing resveratrol (0, 15, 30 and 60 mg/kg diets) for 10 days. Values are expressed as mean ± standard error of mean (SEM) of 30 flies/vial, 5 replicates per treatment group. (a) Significant difference from control; (b) significant difference from PD flies.

After 21 days, untreated *parkin* mutant (PD) flies had a 75% decrease in survival rate compared with the wild type *w*^1118^ flies (Control, Fig. 1). However, the PD flies fed with diet-containing resveratrol (15, 30 and 60 mg/kg diet) had significant increases in survival rates compared with the untreated PD flies.

### Resveratrol improves climbing activity of *parkin* mutant *D. melanogaster*

A significant reduction in climbing rate was observed in PD flies compared with PD flies treated with resveratrol. The PD flies exposed to resveratrol showed a significant increase in climbing activity when compared with untreated PD flies (p < 0.05; Fig. 1B). Also, a significant reduction in the activity of acetylcholinesterase (AChE) by 3.4-fold was observed in untreated PD flies compared with control. However, the treatment of PD flies with resveratrol improved AChE activity compared with untreated PD flies (approx. 2.1, 2.4 and 2.5 folds for 15, 30 and 60 mg/kg diet of resveratrol, respectively p < 0.05), but not up to the level of the control flies (Fig. 1C).

### Resveratrol alleviates oxidative stress and inflammatory markers in *parkin *mutant flies after 10 days of treatment

**Figure 2:**
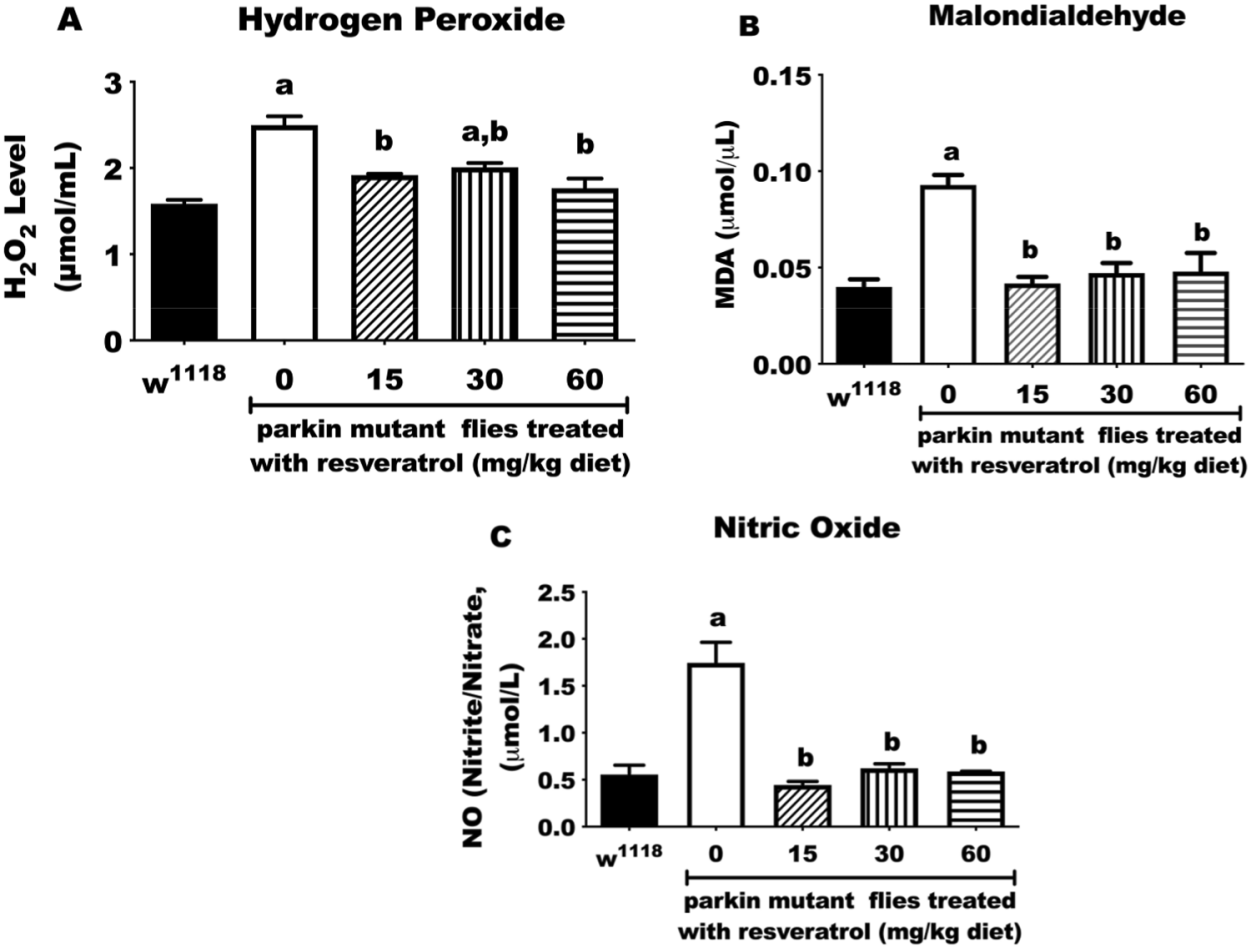
Resveratrol alleviates oxidative stress and inflammatory markers in PD flies after 10 days of treatment. **(A)** Hydrogen peroxide, **(B)** lipid peroxidation and **(C)** nitric oxide (nitrites/nitrates) of control and PD flies fed with diets-containing resveratrol (0, 15, 30 and 60 mg/kg diets). Values are expressed as mean ± SEM of 30 flies/vial, 5 replicates per group. (a**)** Significant difference from control; (b) significant difference from untreated PD flies.

Oxidative stress makers (hydrogen peroxide (H_2_O_2_) and malondialdehyde (MDA)) as well as inflammatory marker (nitric oxide (NO)) may be detrimental to tissues when produced in excessive amounts^27^. We found that PD flies displayed significantly elevated levels of H_2_O_2_, MDA and NO (measured as nitrates/nitrites) (p < 0.05, Fig. 2A–C, respectively) compared with the control group. However, treatment of PD flies with diet-containing resveratrol significantly reduced H_2_O_2_, MDA and NO levels compared with untreated PD flies (p < 0.05; Fig. 2).

### Resveratrol ameliorates reduced levels of non-protein thiols and total thiols displayed in *parkin* mutant *D. melanogaster*

**Figure 3:**
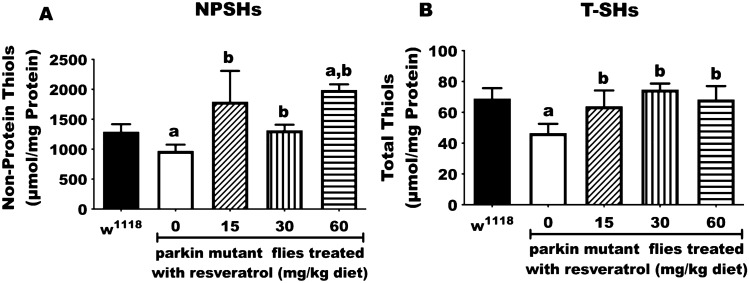
Resveratrol ameliorates reduced levels of non-protein thiols and total thiols of *parkin* mutant *D. melanogaster.* **(A)** Non-protein thiols and **(B)** total thiols of control and *parkin* mutant *D. melanogaster* treated with resveratrol (0, 15, 30 and 60 mg/kg diets) for 10 days. Values are expressed as mean ± SEM of 30 flies/vial, 5 replicates per treatment group. (a) Significant difference from control; (b) significant difference from untreated PD flies.

The thiol system consists of glutathione and other cysteine-containing molecules^28^ that function to maintain the redox balance in biological system^29^. The PD flies displayed significantly reduced levels of non-protein thiols (Fig. 3A) and total thiols (Fig. 3B) by 1.32 and 1.48 folds, respectively, compared with the control group. However, resveratrol significantly increased the levels of non-protein thiols and total thiol compared with untreated PD flies (p < 0.05; Fig. 3).

### Resveratrol mitigates alterations of catalase and glutathione-S-transferase (GST) activities in *parkin* mutant *D. melanogaster*

**Figure 4:**
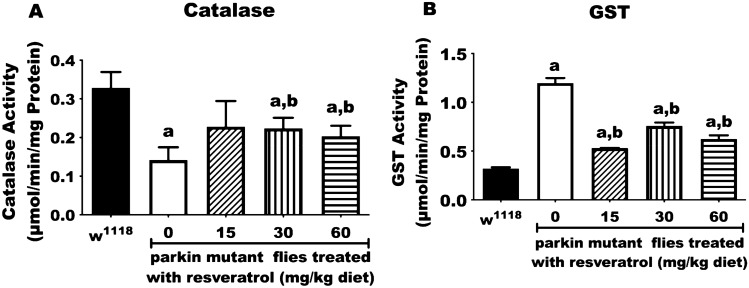
Resveratrol mitigates alterations of catalase and Glutathione-S-Transferase activities in *parkin* mutant *D. melanogaster.* **(A)** Catalase activity and **(B)** glutathione S-transferase activity of control and PD flies exposed to resveratrol (0, 15, 30 and 60 mg/kg diets) for 10 days. Values are expressed as mean ± SEM of 30 flies/vial, 5 replicates per treatment group. (a) Significant difference from control; (b) significant difference from untreated PD flies.

In healthy state, there is a balance between oxidative stress and antioxidant markers^30^. Thus, we determined the activities of two antioxidant enzymes, catalase and GST in control and PD flies. We observed that PD flies displayed significantly reduced catalase activity (Fig. 4A) and elevated activity of GST (Fig. 4B) compared with the control flies. The treatment of PD flies with resveratrol improved catalase and GST activities compared with untreated PD flies, but not up to the level of the control flies (Fig. 4).

### Resveratrol prevents down-regulation of *ple *and *Sod1* genes in *parkin *mutant *D. melanogaster*

**Figure 5:**
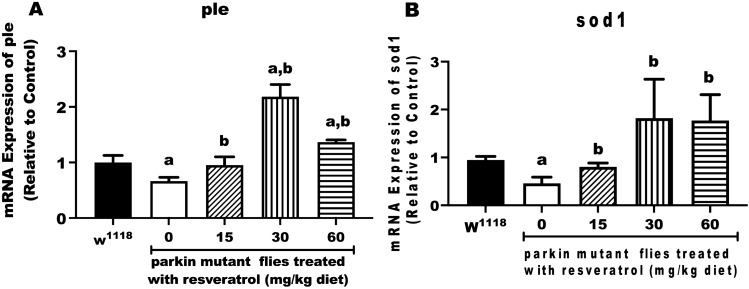
Resveratrol prevents down-regulation of *ple* and *Sod1* in *parkin* mutant *D. melanogaster.* **(A)** mRNA level of *ple* and **(B)** mRNA level of *Sod1* of control and PD flies treated with resveratrol (0, 15, 30 and 60 mg/kg diets) for 10 days. Values are expressed as mean ± SEM. (a) Significant difference from control; (b) significant difference from untreated PD flies.

In order to understand the action of resveratrol at the molecular level, we evaluated its effects on *ple* and *Sod1*. The *ple* encodes tyrosine hydroxylase, the enzyme that catalyzes the first-rate limiting step in dopamine biosynthesis^31^. The *Sod1* encodes superoxide dismutase1 (SOD1)^32^, the enzyme that catalyzes the dismutation of superoxide radical to hydrogen peroxide. The effects of resveratrol (15, 30 and 60 mg/kg diet) on mRNA expression of *ple* and *Sod1* in PD flies are shown in Fig. 5. The PD flies displayed significant reduction in mRNA levels of *ple* and *Sod1* compared with the control. However, resveratrol enhanced mRNA levels of *ple* and *Sod1* in PD flies, with 15 mg/kg diet of resveratrol showing the highest ameliorative effects on *ple* and *Sod1* expressions.

### Histology of control and *parkin* mutant fly brains

**Figure 6:**
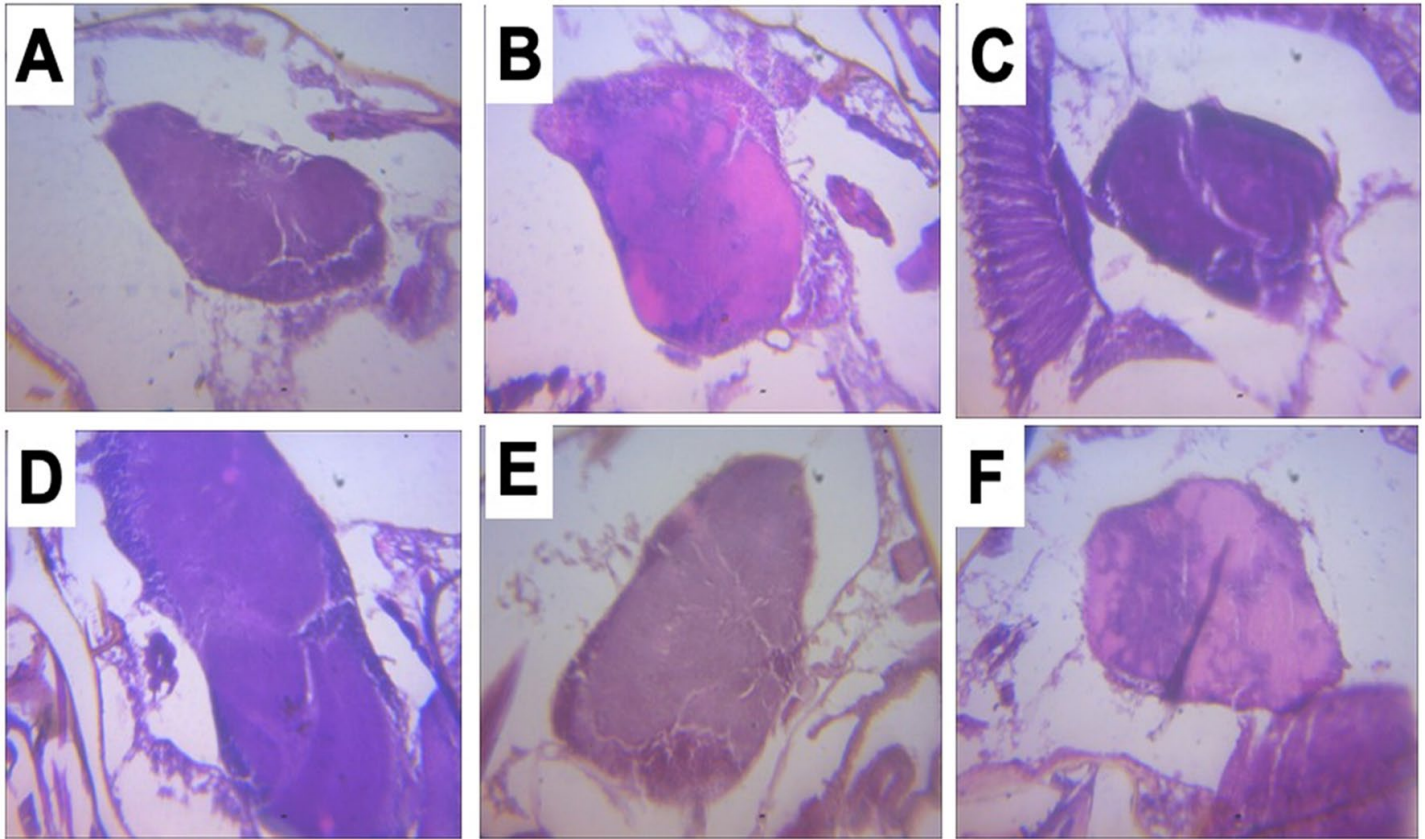
Histology of control and PD fly brains after exposure to resveratrol. (**A)** Control, (**B)** untreated PD fly, (**C)** PD fly treated with resveratrol (15 mg/kg diet), (**D)** PD fly treated with resveratrol (30 mg/kg diet) and (**E)** PD fly treated with resveratrol (60 mg/kg/diet) for 10 days.

The histology data in Fig. 6 indicated no detectable lesion in the brains of control as well as PD flies treated with diets containing resveratrol after 10 days of treatment (Fig. 6).

### Action of resveratrol on mitochondrial fluorescence intensity in *parkin* mutant *D. melanogaster*

**Figure 7:**
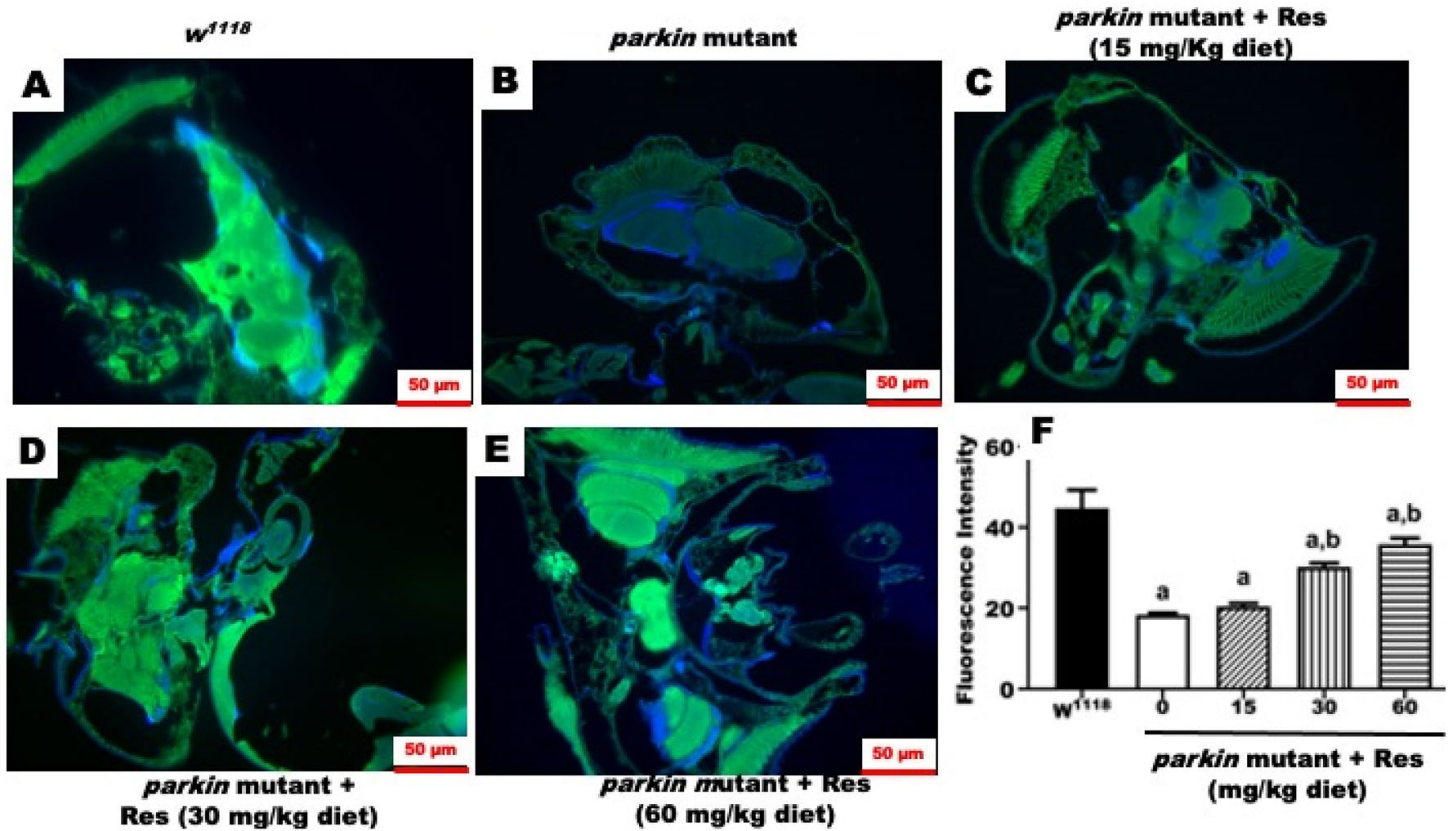
MitoTracker staining of brains of control and PD flies**. (A)** Control brain, (**B)** untreated PD fly brain, (**C) **PD fly brain treated with resveratrol (15 mg/kg diet), (**D)** PD fly brain treated with resveratrol (30 mg/kg diet), (**E**) PD fly brain treated with resveratrol (60 mg/kg/diet) for 10 days and (**F)** mitochondrial fluorescence intensity. (a) Significant difference from control; **(b)** significant difference from untreated PD flies. DAPI: blue; MitoTracker: green.

To understand the action of resveratrol on mitochondrial abundance, the brains were stained with MitoTracker Green (Invitrogen™) as shown in Fig. 7. MitoTracker is a mitochondrial selective probe that covalently binds to mitochondrial proteins via reaction with cysteine residues. This assay is independent of mitochondrial membrane potential and mainly used to represent mitochondrial mass^33^. We found that mitochondrial mass, indicated by fluorescence intensity of MitoTracker Green, was markedly reduced in the brain of PD flies (Fig. 7). However, the treatment of PD flies with resveratrol (30 and 60 mg/kg diet) showed significant increase in mitochondrial fluorescence intensity compared with untreated PD flies (Fig. 7).

## Discussion

Mutations in *parkin* have been established as one of the causes of PD^34^. Parkin is an E3 ubiquitin-protein ligase and a member of the quality control protein system. Available PD treatments, only provide symptomatic reliefs, but could not reduce, reverse or halt the neurodegenerative process^35^. Since oxidative stress is linked with the pathogenesis of PD^36^, any agent that can maintain the redox and oxidative stress-antioxidant balance might be a promising therapeutic drug for PD^37^. Resveratrol (3,5,4’-trihydroxystilbene) is a naturally existing polyphenolic compound found in nuts and fruits such grapes^38,39^. It has been reported to possess both anti-inflammatory and antioxidative properties^40^. It also possesses neuroprotective capacity as previously reported^27^. Here, we carried out different experiments to ascertain if resveratrol could alleviate oxidative stress associated with *parkin* loss-of-function in *D. melanogaster*.

In this study, resveratrol improved the survival rate of *parkin* mutant flies. Different authors have reported the life span-prolonging effect of resveratrol in animal models, including vertebrates (rodents), yeast, and invertebrates (nematodes and insects)^41^. Indeed, resveratrol has been shown to act via different targets such as mediation of autophagocytic death, decreased oxidative stress^41^, activation of sirtuins^42^, reduced inflammation^43^ and lipofuscin formation^44^. The observation that resveratrol increased the survival rate of *parkin* mutant flies aligns with our previous study where resveratrol extended the lifespan of *D. melanogaste*^27^, and that of Valenzano et al.^45^.

Acetylcholine plays important roles in movement and balance^46^. Thus, reduction in its activity may impair acetylcholine function which includes movement. The PD flies displayed reduced locomotor (climbing) activity and acetylcholinesterase activity, which imply dysfunctional cholinergic transmission system. Further, the fact that resveratrol improved locomotor activity and acetylcholinesterase activity in PD flies further indicated its neuroprotective property.

Phase II drug metabolizing enzymes are the major detoxification enzymes that perform important roles in cellular defense. Glutathione-S-transferase (GST), a phase II drug metabolizing enzyme, participates in the detoxification of xenobiotics via conjugation with glutathione^47^. Indeed, overexpression of GST activity has been shown to be protective in *parkin* mutant fly model^48^. In this study, *parkin* mutant flies showed increased GST activity, which might be an adaptive response to *parkin* mutation. In addition, the levels of non-protein thiols and total thiols were reduced in *parkin* mutant flies. Interestingly, these markers were improved when PD flies were fed with diet-containing resveratrol. The thiol system consists of glutathione and other cysteine-containing molecules^28^ that function to maintain the redox balance in biological system^29^. Glutathione acts as an antioxidant and it also acts as a vital factor in the detoxification of xenobiotics together with GST. Thus, the reduction in the levels of these thiols suggest redox imbalance elicited by *parkin* mutation in *D. melanogaster.* The observed increase in the level of non-protein thiols in PD flies treated with resveratrol (15 and 30 mg/kg diet) might be an adaptive protective response due to *parkin* mutation.

Catalase is a first-line antioxidant defence enzyme that catalyzes the conversion of H_2_O_2_ to water and molecular oxygen. Since catalase is required for the breakdown of H_2_O_2_, an inhibition of its activity would be expected to result in increased accumulation of H_2_O_2_. The loss of function of Parkin protein caused an overload of reactive oxygen species, including H_2_O_2_, as a result of defective mitophagy. In PD, oxidative stress is deleterious due to accumulation of H_2_O_2_^49^. Our results showed an increased level of H_2_O_2_ in the *parkin* mutant flies, indicating oxidative stress. The observation that resveratrol reduced H_2_O_2_ level, implies that oxidative stress in the flies was minimised.

Nitric oxide is a gaseous and easily diffusible molecule that acts as neurotransmitter in synapses^50^. Above physiological level, it can act as a reactive nitrogen species and induce oxidative stress in tissues. It forms secondary intermediates called peroxynitrites, which react with macromolecules, causing nitrosative damage. Since the brain has the highest amounts of saturated fats, it is a target for oxidation, nitration and peroxidation^51^. S-nitrosylation of *Parkin* produces a loss of function of the protein contributing to PD^52^. An elevation of nitrates/nitrites with increased lipid peroxidation as observed in our study, suggests oxidative damage. The observation that resveratrol ameliorated increased nitric oxide level displayed in PD flies further confirms its antioxidative activity.

In *D. melanogaster, ple* encodes tyrosine hydroxylase, the rate-limiting step in Dopamine (DA) synthesis^31^. In mammals, dopamine is mainly associated with motor control and reward. In flies, dopamine modulates a range of behaviours such as locomotion, learning, sleep and courtship^53^. As found in mammals, flies synthesize DA from the aromatic amino acid, tyrosine, via the coordinated action of tyrosine hydroxylase and DOPA decarboxylase^54^. Our data revealed the downregulation of *ple* in PD flies compared with the control flies. This might imply a consequent reduction in the synthesis of DA, which is the hallmark of PD. Resveratrol offered protective action by upregulating *ple* with maximum rescue effect at 15 mg/kg diet concentration. This result thus revealed the neuroprotective action of resveratrol (15 mg/kg diet) with respect to *ple* expression*,* which might consequently lead to increase in DA synthesis.

Superoxide dismutase 1 (SOD1), encoded by *Sod1* in flies*,* plays important function in the antioxidant system^32^. It offers protection against ROS by breaking down superoxide radicals to oxygen and H_2_O_2_, thereby making the latter susceptible to catalase degradation. This consequently prevents oxidative stress that can predispose to different diseases such as PD^55^. Thus, a reduction in the mRNA level of *Sod1* noted in the PD flies further implies that oxidative oxidative stress accompanied *parkin* mutation. This further explains the reason for the accumulation of H_2_O_2_ observed in this study. Conversely, treatment of PD flies with resveratrol (15 mg/kg diet) restored mRNA level of *Sod1,* which might imply increase in the activity of SOD1 compared with PD flies, leading to the breakdown of excess superoxide radicals in the flies.

There was no detectable histological lesion in the brains of PD flies treated with resveratrol. Thus, we evaluated mitochondrial abundance in the brains of flies using MitoTracker Green, which is commonly used to stain mitochondria^56^. It binds covalently to sulfhydryl groups of mitochondrial proteins^33^. There was reduction in mitochondrial intensity in the brains of PD flies compared with control flies. Interestingly, resveratrol significantly increased mitochondria intensity in the brains of PD flies.

**Figure 8:**
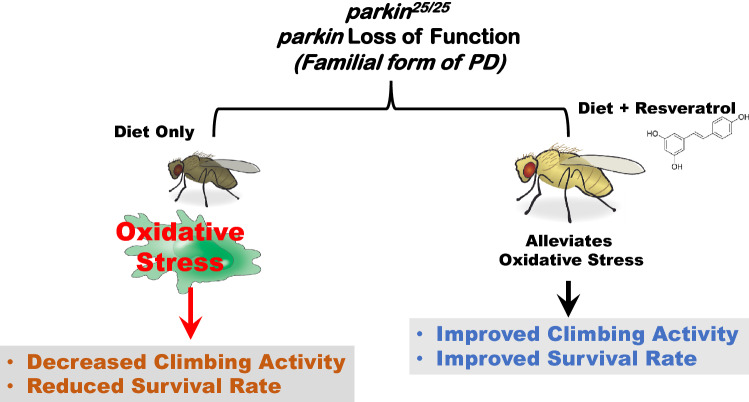
Rescue mechanisms of resveratrol in *parkin* mutant *Drosophila melanogaster* model of Parkinson’s disease. Resveratrol alleviates oxidative stress, improved survival rate and climbing activity in PD flies. File: Drosophila-drawing.svg—Wikimedia Commons, (n.d.). https://commons.wikimedia.org/wiki/File:Drosophila-drawing.svg (accessed February 21, 2022).

Taken together, resveratrol improved survival rate and locomotor performance of PD flies. It maintains oxidant-antioxidant homeostasis and prevented down regulation of *ple* and *Sod1* genes in PD flies (Fig. 8). We did not investigate the reason(s) for the apparent lack of concentration-dependent correlations of resveratrol in the levels of thiols, activities of catalase and GST as well as mRNA levels of *ple* and *Sod1* genes in this study. Nevertheless, this study indicated that appropriate concentrations of resveratrol can reduce oxidative stress associated with *parkin* loss-of-function mutation and therefore might be harnessed for the management of PD. It would be interesting to understand if resveratrol could rescue other forms of genetic-induced models of PD.

## Materials and methods

### Chemicals

All chemicals used in this study were commercial analytical grade products. Reveratrol 98% (HPLC) was purchased from AK Scientific, 30,023 Ahern Ave, Union City, CA 94587, USA.

### Fly stocks

The *D. melanogaster* strain *w; parkin*[25]/TM6B.GFP^12^ and wildtype *w*^*1118*^ were the flies previously used in Dr. Alexander Whitworth’s laboratory, MRC Mitochondrial Biology Unit, University of Cambridge, UK. The flies were maintained at temperature 25 ± 2 °C and 60% relative humidity, under 12 h dark/light cycle in the *Drosophila* laboratory, Department of Biochemistry, Faculty of Basic Medical Science, College of Medicine, University of Ibadan, Ibadan, Nigeria. The *Drosophila* medium was composed of cornmeal medium, brewer's yeast, agar–agar and nipagin (preservative).

### Generation of *parkin* mutant (PD) *D. melanogaster*

Male and female, *w; parkin*^*25*^/TM6B.GFP, flies were allowed to mate in vials containing diet for 3 days and removed. Then, larvae and pupae were followed up to the adult stage, and the pupae without tubby body were noted. Following eclosion, the *w*; *parkin*^*25*^*/parkin*^*25*^ (PD) flies were separated, reared under controlled environmental conditions and monitored for 72 h before further studies. Thereafter, they were carefully selected, under brief CO_2_ anesthesia, 3 days after eclosion. We used the *w*^*1118*^ flies with white eyes (the strain from which the *parkin*^*25*^*/parkin*^*25*^ flies were generated) as control.

### Treatment of PD flies with resveratrol

The control flies were treated with diet-containing vehicle (ethanol, 2% final concentration), while the PD flies were treated with resveratrol: 15 mg/kg diet (0.15 mg/10 g diet, approximately 6.57 mM); 30 mg/kg diet (0.30 mg/10 g diet, approximately, 13.14 mM) and 60 mg/kg diet (0.60 mg/10 g diet, approximately, 26.28 mM), for 21 days to evaluate survival rate. These concentrations were selected based on our previous study involving treatment of wild-type *D. melanogaster* with different concentrations of resveratrol (7.5, 15.0, 30.0, 60.0 and 120.0 mg/kg diet), in which 15, 30 and 60 mg/kg diets of resveratrol extended lifespan by 20.9, 39.5 and 41.86%, respectively^27^.

### Survival rate analysis

Survival rate was carried out to determine the appropriate concentrations and duration of exposure of flies to resveratrol. Thus, 1-to 3-days old control and PD flies were divided into different groups of 30 flies each in 5 replicates. The survival study was carried out by recording the number of dead flies daily for the entire exposure period (21 days). The diet mixed with resveratrol was changed every 5 days. At the end of the treatment, data was analyzed and plotted as percent of live flies^57^. Kaplan–Meier curves were plotted and log-rank tests performed. Based on the data obtained, 10 days duration of treatment and concentrations of resveratrol (15.0, 30.0 and 60.0 mg/kg diet) were chosen for subsequent treatments to evaluate the rescue role of resveratrol in PD flies by carrying out different behavioural, oxidative stress and antioxidant markers as well as mRNA expression of *ple* and *Sod1 genes*.

### Preparation of sample for biochemical assays

Control and PD flies of age 1-to 3- days old were divided into different groups of 30 flies each in 5 replicates and treated with resveratrol (15.0, 30.0 and 60.0 mg/kg diet) for 10 days. Thereafter, they were anaesthetized under CO_2_, weighed and homogenized in 0.1 M phosphate buffer, pH 7.0 (ratio of 1 mg: 10 μL), and centrifuged at 4000 × *g* for 10 min at 4 °C. The supernatants were then separated from pellets into labelled clean microfuge tubes and used for the analyses of oxidative stress, antioxidant and inflammatory markers.

### Behavioural assay

This was carried out manually using negative geotaxis/locomotor assay^58^. Briefly, ten flies from control and PD groups were briefly anaesthetized using ice and placed in glass column (15 cm length and 1.5 cm in diameter). After recovery from anaesthesia, they were gently tapped to the bottom of the column. Then, the number of flies that climbed to the 6 cm mark were recorded. The data were then expressed as percentage of flies that crossed up to and beyond the 6 cm mark of the column.

### Determination of total thiols and non-protein thiol content

Total thiol content was assayed by the method of Ellman^59^. Briefly, the reaction mixture contained 170 μL of 0.1 M potassium phosphate buffer (pH 7.4), 20 μL of sample, and 10 μL of 5,5′-dithiobis-(2-nitrobenzoic acid (DTNB). After incubation for 30 min at room temperature, the absorbance was measured at 412 nm using SpectraMax microplate reader (Molecular Devices). For non-protein thiol, the sample was precipitated with 4% sulphosalicyclic acid (4%) in the ratio of 1:1. The samples were kept at 4 °C for 1 h and then subjected to centrifugation at 5000 rpm for 10 min at 4 °C. The assay mixture consisted of 170 µl of 0.1 M phosphate buffer, 20 µl of supernatant and 10 µl of DTNB. The reaction was allowed to incubate for 30 min at room temperature, and the absorbance was read at 412 nm using SpectraMax microplate reader. For both total thiols and non-protein thiols, reduced Glutathione (GSH) was used as standard, and the data were expressed as in μmol/mg of protein.

### Determination of glutathione-S-transferase activity

Glutathione-S-transferase activity was determined using the method of Habig and Jacoby^60^ using 1-chloro-2,4-dinitrobenzene (CDNB) as substrate. The assay mixture was made up of 270 μL of solution A (20 mL of 0.25 M potassium phosphate buffer, pH 7.0, with 2.5 mM EDTA, 10.5 mL of distilled water and 500 μL of 0.1 M GSH at 25 °C), 20 μL of the sample (1:5 dilution), and 10 μL of 25 mM CDNB. The reaction mixture was monitored at 340 nm for 5 min at 10 s intervals in a SpectraMax microplate reader (Molecular Devices). The results were expressed as μmol/mins/mg protein.

### Determination of catalase activity

Catalase activity was determined based on the method of Aebi^61^. Briefly, the H_2_O_2_ clearance was monitored at 240 nm, for 2 min (10 s intervals) and at 25 °C with a UV/Visible spectrophotometer. The reaction assay contained 1800 μL of 50 mM phosphate buffer (pH 7.0), 180 μL of 300 mM H_2_O_2_, and 20 μL of sample (1:50 dilution). The loss in absorbance of H_2_O_2_ was monitored for 2 min at 240 nm and thereafter used to calculate catalase activity expressed as μmol of H_2_O_2_ consumed/mins/mgprotein.

### Determination of acetylcholinesterase (AChE) activity

The method of Ellman et al.^62^ was used to determine the activity of AChE. The assay mixture contained 135 μL of distilled water, 20 μL of 100 mM potassium phosphate buffer (pH 7.4), 10 mM DTNB, 5 μL of sample and 20 μL of 8 mM acetylthiocholine as initiator. The reaction was then monitored for 5 min at 15 s intervals at 412 nm using a SpectraMax microplate reader (Molecular devices). The enzyme activity was estimated as μmol of acetylthiocholine hydrolyzed/min/mg protein.

### Hydrogen peroxide generation

Hydrogen peroxide level was determined according to the method of Wolff^63^. The assay mixture contained 590 μl of FOX1 (Ferrous Oxidation-Xylenol orange) reagent and 10 μl of sample. This was followed by 30 min incubation at room temperature, and the absorbance measured at 560 nm. The concentration of the hydrogen peroxide generated was determined using extinction coefficient of H_2_O_2_ and expressed as μmol/ml.

### Protein determination

The method of Lowry et al.^64^ was used to determine total protein content using Bovine Serum Albumin (BSA) as a standard.

### Determination of nitric oxide level

The nitric oxide (nitrate/nitrite) level was determined using Griess reaction method^65^. The fly homogenate was incubated with Griess reagent at room temperature for 20 min, and the absorbance was read at 550 nm. The concentration of NO in the sample was calculated using the standard calibration curve of NaNO_2_^65^ and expressed as μmol/L.

### Lipid peroxidation assay

The assay was performed as described by Ohkawa et al.^66^. The reaction mixture was made by adding 5 µL of 10 mM butylhydroxytoluene, 200 µL of 0.67% thiobarbituric acid, 600 µL of 1% *O*-phosphoric acid, 105 µL distilled water, and 90 µL whole fly homogenate. The resultant mixture was incubated at 90 °C for 45 min, and the OD was measured at 535 nm. The results were expressed as μmol of malondialdehyde (MDA) formed/μL.

### The isolation of RNA and quantitative real- time RT-PCR

Total RNA was isolated from 25 mg whole flies using Trizol (TRI Reagent^®^, Zymol Research) using manufacturer’s protocol as previously described^67^. The RNA isolated was resuspended in 50 µl RNase-free water, quantified spectrophotometrically using MaestroNanoDrop Pro (Maestro Gen) and visualized in 2% agarose gel after gel electrophoresis (BioRad). Total RNA (0.3 µg) was used for cDNA synthesis following the manufacturer’s protocol for ProtoScript II kit (New Egland BioLabs) and carried out in BioRad T100 thermal cycler. The primer sequences used in this study (*ple* and *Sod1*, Table 1) were obtained from the GeneBank overview (GenBank Overview (nih.gov)) and designed using the primer BLAST tool (https://www.ncbi.nlm.nih.gov/tools/primer-blast/) which were custom synthesized by Invitrogen. Subsequently, qPCR was carried out and the mRNA expression levels were standardized to two genes (Beta Tubulin 86D and RPL32). Luna Universal qPCR Master mix kit was used for qPCR. A total reaction volume of 20 µl was used with 9 ng of cDNA, Luna Universal qPCR Mix, 10 µM forward and reverse primers, and nuclease-free water. The cycling condition was as follows: an initial denaturation at 95 °C for 60 s followed by 40 cycles of 95 °C for 15 s, 60 °C for 60 s followed by a dissociation curve analysis. The SYBR fluorescence was analysed by the SDS 2.1 software (Applied Biosystems, ABI Prisms 7900HT). The reaction was carried out in triplicates of each independent group. Dissociation curves at 60–95 °C was carried out to ascertain the amplification of a single specific product for each reaction. The 2^−ΔΔCT^ method was used to determine the expression values of each gene.

**Table 1:** Sequence of primers.

Genes	Forward sequence	Reverse sequence
Β-Tubulin	TGGGCCCGTCTGGACCACAA	TCGCCGTCACCGGAGTCCAT
RPL32	CCCAAGATCGTGAAGAAGCG	TGGGCTTGCGCCATTTGTG
*ple*	CAGCAAGGCAAATGATTACGGT	AATCCGGGGTGGTTCATGTC
*Sod1*	GGAGTCGGTGATGTTGACCT	GTTCGGTGACAACACCAATG

### Histology of fly brains

Adult fly brains were fixed in 10% neutral buffered formalin, deparaffinized and processed for Hematoxylin and Eosin (H/E) histological staining^68^. The slides were viewed using light microscopy and interpreted by Veterinary Pathologists who were blinded to the control and PD fly brains.

### Mitochondrial staining in fly brains

Apart from H/E histological study, we carried out additional experiment to evaluate mitochondrial mass/abundance (based on fluorescence intensity of MitoTracker™ Green stain, Invitrogen™)^56^ in the brains of control and *parkin* mutant flies treated with resveratrol for 10 days. Briefly, the fly brains (15/group) were excised, fixed in 4% buffered formalin for 40 min and transferred to 30% sucrose solution in PBS. Thereafter, the whole brains in a group were dehydrated and embedded in paraffin. Then, 5 µm sections of fly brains were prepared from a block and stained on charged slides. The slides were deparaffinised in xylene solutions (10 min each) and rehydrated in decreasing concentrations of ethanol solution in PBS. The slides were then stained with 0.1% MitoTracker™ Green (Invitrogen™) in PBS for 20 min. Nuclei were stained with DAPI (0.1 µg/mL) and slides were mounted in PBS-Glycerol (1:1) solution containing N-propyl gallate. Images were acquired using Zeiss Axioscop fluorescent microscope and signal intensity quantitation as well as background correction were carried out using ImageJ.

### Statistical analysis

For statistical analysis, GraphPad Prism 9 was used. The Kaplan–Meier’s method was used to analyze the survival rate and comparisons were made with the log-rank tests. For biochemical analyses, statistical significance was evaluated using one way analysis of variance (ANOVA), followed by Dunnett’s post hoc test. Data points correspond to the mean of independent experiments and error bars (S.E.M); the level of significance was set at *p* < 0.05 and indicated in the charts.

## Data Availability

All data that support the findings of this study are available on reasonable request to the corresponding author. The contributing authors declare that all relevant data are included in the paper.
